# Reactive Extrusion of Sorghum Flour with Ozone Modifies the Texture, Thermal Behavior, and Digestibility of Starch and Proteins

**DOI:** 10.3390/foods15020375

**Published:** 2026-01-20

**Authors:** Pablo Palavecino, Esteban Carrillo Parra, Marianela Rodriguez, María Isabel Curti, Mariela Bustos Shmidt, Pablo Ribotta

**Affiliations:** 1 Facultad de Ciencias Exactas, Físicas y Naturales, Universidad Nacional de Córdoba, Córdoba 5000, Córdoba, Argentina; ejocarrillo@mi.unc.edu.ar (E.C.P.); pribotta@agro.unc.edu.ar (P.R.); 2 Instituto de Ciencia y Tecnología de los Alimentos Córdoba (ICyTAC), CONICET—UNC, Córdoba 5000, Córdoba, Argentina; marianelarodriguez@agro.unc.edu.ar (M.R.); mbustos@agro.unc.edu.ar (M.B.S.); 3 Facultad Ciencias Exactas y Naturales, Universidad Nacional de La Pampa, Santa Rosa 6300, La Pampa, Argentina; mariacurti@gmail.com; 4 Instituto de Ciencias de la Tierra y Ambientales de La Pampa (INCITAP), CONICET—UNLPAM, Santa Rosa 6300, La Pampa, Argentina

**Keywords:** cereal flour, pasting profile, extrudates, in vitro digestion

## Abstract

This study addresses the need for sustainable and clean-label processing methods to enhance the functional and nutritional properties of sorghum flour. Reactive extrusion combining high shear forces and ozonization was selected as an environmentally friendly modification strategy. Whole and polished sorghum flours were processed using a twin-screw extruder, with ozone introduced via ozonated feed water under varying temperature profiles (140 °C and 160 °C) and moisture contents (20% and 23%). Characterization included specific mechanical energy (SME), textural attributes, water absorption and solubility indices (WAI/WSI), viscosity profiles (RVA), and surface chemistry via X-ray photoelectron spectroscopy (XPS). Finally, in vitro digestion was used to monitor the kinetics of starch and protein hydrolysis. Ozone reduced SME, increased extrudate density, and lowered expansion and fracture force, particularly in polished flour. The XPS confirmed successful oxidation, showing the conversion of hydroxyl groups into carbonyl and carboxyl groups. Ozone also improved water absorption but reduced solubility and decreased viscosity parameters in polished flour. In vitro digestion showed that extrusion ozonation enhanced protein digestibility at ~25%. At the gastric phase, ozonized whole samples showed 18.3% starch hydrolysis, and ozonized polished flour showed 8.3%, whereas non-ozonized flours exhibited ~25%. These findings prove that ozone-assisted reactive extrusion differentially changes sorghum flour properties, offering a promising approach for improved food applications.

## 1. Introduction

The increasing global demand for sustainable food sources and ingredients for specialized diets positions sorghum (*Sorghum bicolor* (L.) Moench) as a crucial and rapidly evolving agricultural commodity. Sorghum is widely recognized for its robust nature, remarkable drought tolerance, and resistance to waterlogging, becoming a major staple crop, particularly across arid and semiarid regions. Sorghum is rich in nutrients, fiber, and bioactive components and is also a gluten-free alternative for individuals suffering from celiac disease or gluten intolerance, leading to its increasing incorporation into commercial products like breads, pastas, and snack foods.

Despite these significant advantages, the full potential of sorghum use is often limited by inherent drawbacks: the presence of antinutritional factors, mainly present in grain external layers, and generally poor protein and starch digestibility. To overcome these limitations, processing and modification strategies are essential to enhance both the nutritional quality and functional characteristics of sorghum flour. Typically, the primary approach involves removing or minimizing the outer layers through grain polishing before milling, allowing for the production of either whole or refined flour [1,2]. Traditional methods used to change flour properties, such as chemical treatments or wet cooking, often involve multiple lengthy processing stages, extensive washing, and high energy costs, which can result in the production of contaminant residues that severely limit industrial scalability.

In contrast, extrusion cooking technology offers a highly efficient, continuous, and versatile modification platform. As a high-temperature, short-time (HTST) process, extrusion is noted for its cost-effectiveness, high productivity, energy efficiency, and low waste generation. Extrusion uniquely integrates several unit operations—including mixing, conveying, kneading, cooking, shaping, and pasteurization—into a single system. The intense thermo–mechanical energy applied during extrusion (high pressure, temperature, and mechanical shear) induces profound physicochemical transformations in flours: starch gelatinization and partial degradation (dextrinization), protein denaturation, and the reduction or elimination of antinutritional factors, such as tannins, phytate, and trypsin inhibitors. To further enhance modification efficacy and address the limitations of conventional chemical treatments, the field has transitioned toward reactive extrusion. Reactive extrusion involves performing chemical or structural modifications simultaneously with the thermal and mechanical forces inside the extruder barrel, often in a single step. This advanced technique combines the process advantages of extrusion with the desired outcomes of chemical modification, minimizing chemical waste and facilitating the production of specialized ingredients [3,4,5].

A promising strategy for functional modification that adheres to user and consumer-friendly demands is the use of ozone. Ozone is generally recognized as safe (GRAS) by the FDA and functions as a powerful oxidant. A key benefit of ozone over traditional chemical agents (e.g., chlorine and hydrogen peroxide) is its rapid decomposition into oxygen, which ensures no harmful chemical residues remain in the food product or the environment. The ozonation of starch leads to significant molecular and functional changes. The oxidation process primarily consists of two reactions: Initially, the substitution of hydroxyl groups in starch molecules with carbonyl and carboxyl groups, which increases the degree of substitution. This step often preferentially occurs in the amorphous region. Subsequently, especially under strong oxidation, glycosidic bonds are broken, leading to the depolymerization of starch molecules [6,7]. Ozone treatment alters protein structure and molecular interactions. Mild treatments promote the oxidation of sulfhydryl groups, leading to the widening of the protein, decreasing surface hydrophobicity, changing both secondary and tertiary structures, and improving in vitro protein digestibility [8,9,10].

Extensive research has detailed the individual effects of modification via extrusion on sorghum flour and the properties of ozone-oxidized starches from various sources (e.g., corn, sago, tapioca, rice and cassava) [1,2,4,6]. Despite the development of reactive extrusion using other modifying agents (such as alkaline hydrogen peroxide), a significant research gap exists concerning the integration of ozone within the extrusion process (reactive extrusion) for modifying sorghum flour [3,5]. The current literature lacks comprehensive data on how this combined, clean-label process simultaneously influences the textural qualities, thermal characteristics, and crucial in vitro digestibility profiles of both starch and proteins in sorghum.

This study aims to establish reactive extrusion with ozone as an innovative method to improve the functional and nutritional properties of modified sorghum flour and extrudates. It specifically examines how this combined process changes the texture, thermal behavior, and in vitro digestibility of starch and proteins in sorghum flour.

## 2. Materials and Methods

### 2.1. Materials

Brown sorghum grains were supplied by Molino Carlos Boero Romano S.A.I.C. (San Francisco, Cordoba, Argentina) and cleaned following the procedure described by Curti et al. [11]. Briefly, grains were sieved, rinsed, and dried in an air-forced oven for 24 h at 40 °C to eliminate contaminants originating from the harvest site.

To prepare polished flour, the dried sorghum grains were treated in an abrasive dehuller (PAZ-1-DTA, Zaccaria, Brazil). In this device, batches of 100 g of grains were processed and maintained inside for 120 s. Whole and polished sorghum grains were ground using a hammer mill equipped with a 1 mm screen (Pulverisette^®^ 16, Fritsch, Germany). Whole sorghum flour (W) and polished sorghum flour (P) were kept in hermetic black plastic containers until usage.

### 2.2. Raw Material Composition

A proximate analysis of both sorghum flours was performed according to the standard methods of AOAC [12] (moisture: method 925.10; ash: method 923.03; lipid: method 920.39 and protein: method 920.87). Total polyphenol content was evaluated through the Folin–Ciocalteu method according to previous sorghum product assays [13]. Total polyphenol content (TPC) was expressed as g gallic acid (GA) per 100 g of extrudate on a dry basis.

### 2.3. Extrusion Cooking Process

A co-rotating twin-screw self-cleaning extruder (Process 11-Hygienic, Thermo Scientific, Berlin, Germany) equipped with eight independently controlled temperature zones was used. The extruder had a length-to-diameter (L/D) ratio of 40:1, and the die diameter was 3 mm. The arrangement of screw elements was the standard three-stage configuration, which included two kneading blocks (recommended settings for cereal flour processing). Flour was dispensed with a screw feeder at a flow rate of 2.05 kg/h. The recorded process variables were average torque, die temperature, and pressure.

Three factors were analyzed at two levels: temperature profile (Table 1), water flow rate, and the use of either ozonated or non-ozonated water. Water flow rates were adjusted to 20 and 23% of moisture in the feeding material and were identified as low (L) and high (H), respectively. To perform the reactive extrusion, ozone was generated from industrial oxygen (95% purity) using an ozone generator unit (custom-designed model, Ozonizer). Ozone was bubbled into distilled water for 10 min at a rate of 36 mg/min and a flow of 1 L/min to achieve the maximum ozone concentration in water (1.5 mg/L). The ozonated water was stored in a bottle with a two-way cap (one connection to a filter and the other to the outlet hose) during the experimental runs.

Extrudates resulting from the extrusion cooking process were dried at 45 °C in a forced air dryer for 24 h. A fraction of dried extrudates was milled using a hammer mill (Pulverisette^®^ 16, Fritsch, Germany) equipped with a 0.75 mm mesh and stored in sealed bags at room temperature until evaluation.

#### Specific Mechanical Energy Input (SME)

Equation (1) [14] was used to calculate the specific mechanical energy input (SME).

(1) $$SME\;(Wh\;{kg}^{-1})=\frac{\frac{n}{n_{max}}\times \frac{M_{d}-M_{d,\;unload}}{100}}{\dot{m}}\times P_{max}$$

where *n* and *n_max_* are the actual and maximum screw speeds (1000 min^−1^), *M_d_* and *M_d,unload_* are the actual and idle torques (%), $\dot{m}$ represents the total mass flow (kg·h^−1^), and *P_max_* is the maximum engine power (1.5 kW).

### 2.4. Extrudate Dimensional Characterization

The extrudate dimensional characterization was determined following the method proposed by Liu [15], with slight modifications. The length and diameter of the extrudates were measured using a caliper. Three extrudates were randomly selected per sample. Length measurements were taken for all three, and diameter measurements were performed at four randomly chosen locations along each extrudate.

The expansion index (EI) was obtained as the ratio between the mean diameter of the extrudate and the die diameter (3 mm). Subsequently, the mass of the extrudates was determined, and the density was calculated as the ratio of mass to volume, expressed in g/cm^3^.

### 2.5. Texture of Extrudate

The fracture force, defined as the maximum force needed to break an extrudate, was measured using a three-point bending test. Ten extrudates from each sample were analyzed using a texture analyzer (Universal Testing Machine model 3342, Instron, Norwood, MA, USA) equipped with a load cell of 500 N and test speed of 50 mm min^−1^. Each piece of 5 cm was perpendicular, placed over the anvils, and symmetrically distributed. At least four replicates were performed for each sample. Bluehill 2^®^ software (Version 2.27) for Windows (Instron, USA) was used for instrument operation and data collection.

### 2.6. Analysis of Flour Properties

#### 2.6.1. X-Ray Photoelectron Scattering (XPS)

The surface chemical composition was assessed using an X-ray photoelectron spectrometer (K-Alpha, Thermo Fisher, Waltham, MA, USA) with an excitation source of Al Kα X-rays (hv = 1486.6 eV), a current of 16 mA, and a voltage of 12 kV. The spot size was 300 µm, where full spectrum data were acquired at a passing energy of 100 eV with a resolution of 0.5 eV, whereas the high-resolution spectrum was obtained at a passing energy of 20 eV with a resolution of 0.05 eV [2]. The XPS spectrum was corrected with C1s = 284.80 eV binding energy as the energy standard. Open-source KherveFitting software (version 1.5) was used to process data.

#### 2.6.2. Water Solubility and Absorption Indexes

Water solubility (WSI) and absorption (WA) indexes of flour were determined following the method of Palavecino et al. [16], with slight modifications. Briefly, 2.0 ± 0.1 g (db) was weighed for each sample in 50 mL centrifuge tubes and was suspended in 25 mL of distilled water. The mixture was vortexed vigorously and then oscillated for 30 min. Subsequently, the samples were centrifuged at 3500 rpm for 20 min and the supernatant was transferred to a Petri dish and dried in an oven at 55 °C with an air velocity of 4 m/s for 24 h. Once dried, the Petri dish was weighed to determine the difference from the empty dish, and the result is expressed as grams of dried supernatant per gram of sample, thereby obtaining the WSI. The pellet was weighed, and the WA was expressed as g of absorbed water by g of sample. Determinations were performed in triplicate and expressed as the mean.

#### 2.6.3. Color

The color of native flours and milled extrudates was determined following the method described by Palavecino et al. [16] with a colorimeter (CM-600d; Konica Minolta, Tokyo, Japan) using a D65 illuminant at 10° observation. A quantity of approximately 3 g of flour was placed on a flat, white surface and covered with 0% reflectance glass. The colorimeter was then positioned at the center of the glass to obtain measurements expressed in the CIELAB scale (L*, a*, b*). The total color difference ΔE was calculated as Equation (2).

(2) $$∆E=\sqrt{{\left(L_{w}^{*}-L_{o}^{*}\right)}^{2}+{\left(a_{w}^{*}-a_{o}^{*}\right)}^{2}+{\left(b_{w}^{*}-b_{o}^{*}\right)}^{2}}$$

where the subscript *W* refers to the sample without ozone treatment and *O* refers to the ozonized ones. This parameter was calculated to assess the effect of the oxidizing power of ozone bleaching extruded flour.

#### 2.6.4. Viscosity Profile

**Figure 1:**
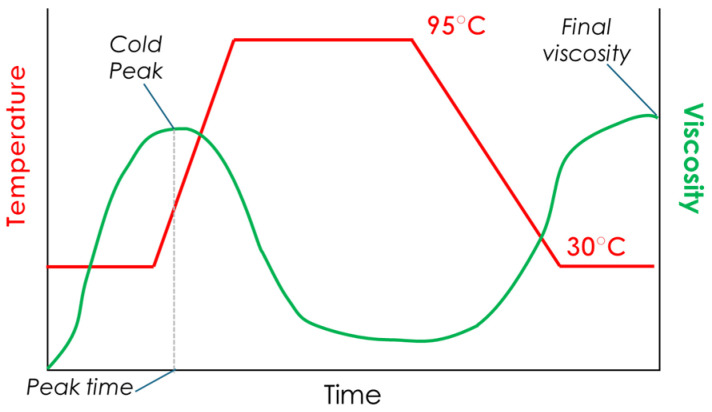
Extrudate flour RVA characteristic pasting profile and main parameters.

The pasting properties were determined using a Rapid Visco Analyzer (RVA 4500, Newport Scientific, Warriewood, Australia) following the pregelatinized starch method, a procedure included in the software Thermocline (version 3.17, Perten, Macquarie Park, Australia), with slight modifications. Briefly, 5.00 ± 0.01 g of flour was weighed in a canister and 2.00 ± 0.01 g of castor sugar was added and thoroughly mixed. Then, 25.0 ± 0.1 mL of cold (15 °C) distilled water was added to the solids. The temperature profile began at 30 °C and was kept for 2 min. The temperature then increased to 95 °C and was held constant for 3 min, after which it decreased back to 30 °C until the conclusion of the assay (Figure 1). The RVA analysis quantifies starch pasting behavior via parameters such as cold peak (CP), final viscosity (FV), and peak time. These metrics assess starch quality and functional properties relevant to food processing applications.

The cold peak (CP) is defined as the maximum viscosity recorded at the onset of the RVA extruded flour test, when the sample is kept at a low temperature. This measurement reflects the starch’s capacity to hydrate under cold conditions. A high CP value suggests that the starch quickly absorbs water, which is characteristic of a high level of starch pre-gelatinization. In native flours, the cold peak corresponds to the viscosity point where most of the starch granules have swollen to their fullest extent while staying structurally intact. This marks the beginning of the gelatinization process. For extruded flours, the CP refers to the cold viscosity peak, as these flours have already undergone starch pre-gelatinization during extrusion, resulting in altered starch granules.

Final viscosity (FV) refers to the capacity of a material to develop a viscous paste or gel after cooking and cooling, and it is considered the primary parameter for assessing the quality of starchy products. Enhanced interactions among amylose molecules or hydrolysates of comparable length led to increased resistance to flow, resulting in higher system viscosity.

### 2.7. In Vitro Digestion Using Static Method

In vitro digestion of the extrudate samples was performed in duplicate according to Bustos et al. [17] to evaluate starch hydrolysis. This method is based in the highly recognized standardized static method proposed by INFOGEST [18]. Briefly, the ratio used was 50/50 *w*/*v* for sample/Simulated Salivary Fluid (SSF), oral content/Simulated Gastric Fluid (SGF), and gastric content/Simulated Intestinal Fluid (SIF) corresponding to three stages: oral, gastric, and intestinal.

#### 2.7.1. Starch Digestibility

During in vitro digestion, aliquots of 1 mL were withdrawn at time 0, after oral digestion, after 120 min of the gastric phase, and at the end of the intestinal step (another 120 min) to monitor the hydrolysis degree of starch and its kinetic parameters. Starch hydrolysis was monitored by an analysis of reducing sugar content in each aliquot using the 3,5 dinitrosalicylic acid (DNS) method.

#### 2.7.2. Quantification of Free-Amino Groups (OPA Method)

Free amino groups in the supernatant from the digests were measured using the o-phthaldialdehyde (OPA) method according to Nielsen et al. [19] and used OPA reagent (P1378, Merck KGaA, Saint Louis, MO, USA) and serine as standard and deionized water for blank value. The results were expressed as absolute values of serine equivalents in 0.5 g of proteins corresponding to digested samples.

### 2.8. Statistical Analysis

Analyses of variance (ANOVA) with multiple comparison tests (DSG, α = 5%) were conducted using InfoStat software (Version 13p), and the artwork was created in Excel (Microsoft 365 version).

## 3. Results and Discussion

### 3.1. Raw Material Composition

Flour composition (Table 2) is one of the main factors that affect the extrusion process and its products. The moisture content is a critical factor in extrusion; it was considerably low compared to those typically reported for sorghum whole grain flour [11]. Values were a consequence of the flour obtention process. The carbohydrate content was greater in polished flour, primarily because the sorghum endosperm consists predominantly of starch. Protein levels were high but within the typical range of sorghum [16]. Although the literature suggests that refining (bran removal) could result in a decrease in protein content due to its higher concentration in the outer grain layers, the slight difference observed in refined flour may be attributable to the specific milling and polishing processes employed [11]. Nevertheless, the polishing process resulted in a significant reduction in lipid content, as previously documented [20]. These levels were particularly lower in sorghum flours in both cases. This hybrid was specifically selected due to its low lipid content, as oxidation can result in undesirable flavors when lipids are present in high concentrations. The results show that whole grain flour has a higher total polyphenol and mineral content than refined flour. This observation aligns with the scientific literature, as these compounds are primarily found in the outer layers of grain [16,20].

### 3.2. Analysis of the Extrusion Process Parameters

The extrusion of sorghum flours shows that specific mechanical energy (SME) is significantly influenced by material composition and process parameters (Table 3). Polished flour, with a higher starch content, required greater SME than whole flour, probably due to its viscous melt behavior. In addition, the capacity of fibers in whole flour to limit starch water absorption and gelatinization may result in reduced energy requirements during processing [21,22]. Ozone treatment reduced the SME in polished flour, though this effect was less pronounced in whole flour. The starch degradation caused by ozone likely explains this difference, as the fibers and lipids present in whole flour may mitigate oxidative damage during extrusion. Temperature profiles played a critical role: low temperatures (140 °C) maximized SME during partial gelatinization. In contrast, higher temperatures (160 °C) reduced SME via starch dextrinization, lowering system viscosity. Water flow rate was inversely correlated (*p* < 0.05) with SME and die pressure as increased moisture plasticized the melt, easing flow resistance.

### 3.3. Extrudate Dimensional and Textural Results

**Figure 2:**
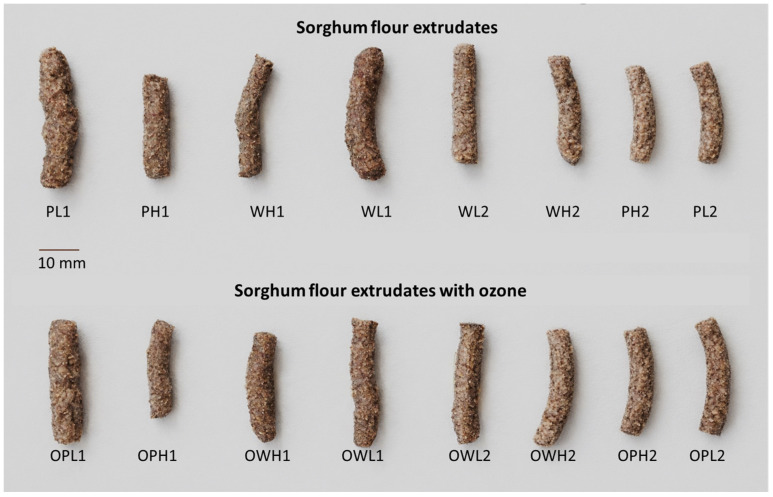
Photograph of extrudates from polished (P) and whole (W) sorghum flours with ozone (O), under low (L) and high (H) water flow rates, and with low (1) and high (2) temperature extrusion profiles.

The expansion index (IE) and density of extruded sorghum flours were strongly influenced by material composition and processing conditions (Figure 2). In general, polished flour extrudates exhibited higher expansion and lower density than whole flour ones, probably due to their higher starch content, helping bubble formation (Table 4). Higher temperatures (160 °C) decreased expansion by promoting excessive dextrinization, whereas lower temperatures optimized expansion by maintaining melting elasticity. Increased water flow reduced extrudate expansion through excessive plasticization, and a lower water content conversely enhanced expansion but resulted in lower density. These results are consistent with previous findings that studied the effect of moisture and extrusion temperature on the expansion index of extrudates of corn, yam, rice, sorghum, and wheatgrass [23,24,25]. The incorporation of ozone into polished sorghum flour resulted in notable changes to both the expansion index and density of the extrudates. This effect can be attributed to oxidative degradation, which increases water interaction within the flour matrix. However, despite the enhanced water absorption, the starch network becomes weakened due to oxidation [10]. As a result, the modified starch matrix is unable to effectively retain the gas generated during the extrusion process. This leads to altered expansion characteristics and structural density in the final extrudate. In the case of whole flour, the application of ozone treatment resulted in only minor changes to the structural properties. This limited effect can be attributed to the stabilizing presence of dietary fiber within the flour matrix. The fiber acts as a reinforcing component, maintaining the overall structure and cohesion of the extrudate even after oxidative modification.

The fracture force of extruded sorghum products was influenced by flour type, ozone treatment, process temperature, and water flow rate (Table 4). Polished flour showed a higher fracture force than whole flour, attributed to its denser starch matrix, whereas whole flour’s fibrous structure reduced structural cohesion. Similar results were reported for corn-extruded snack products enriched with rice bran [26], which is a classic and well-documented effect in cereal science. Bran particles (rich in insoluble fiber, ash, and lipids) act as physical discontinuities within the continuous starch–protein matrix that forms during extrusion. They interrupt the formation of a uniform, strong melt, creating weak points that crack under lower force. In addition, bran reduces the concentration of starch, the primary polymer responsible for forming the viscoelastic melt and the expanded, crisp structure upon exiting the die [4].

Ozonation further weakened the texture, an effect most pronounced in polished flour, as starch degradation and diminished polymer interactions compromised its cohesive matrix. Higher extrusion temperatures (e.g., 160 °C) decreased fracture force by promoting more starch dextrinization and matrix disruption, while lower temperatures (110–140 °C) preserved structural integrity. Increased water flow rate softened the extrudates by enhancing gelatinization and reducing melting viscosity, leading to lower fracture resistance. These are common effects of excessive oxidation, which causes depolymerization and changes to gelatinization temperature and enthalpy [6]. Notably, ozone-modified polished flour extruded at elevated temperatures and water flow rates yielded the weakest textures. Whole flour maintained moderate firmness due to fiber reinforcement. These findings prove that texture can be tailored by adjusting flour composition, oxidative modification, and extrusion parameters, with implications for product applications requiring specific mechanical properties.

### 3.4. Modified Flour Results

#### 3.4.1. X-Ray Photoelectron Scattering (XPS)

**Figure 3:**
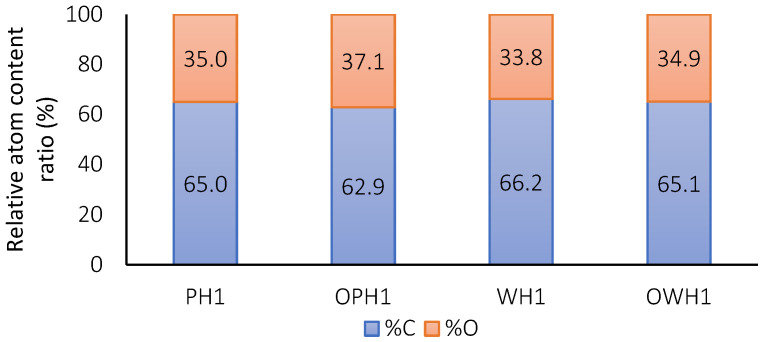
Relative atom content determined by XPS of extrudates from polished (P) and whole (W) sorghum flours.

X-ray photoelectron spectroscopy (XPS) was conducted to confirm the structural changes on the samples by evaluating the chemical environments of carbon (C) and oxygen (O) atoms. The relative abundance of C and O elements in treated flours without oxidation showed a slight difference between whole and polished flours (Figure 3). Proteins and lipids inherently exhibit higher carbon content than starch [27], and because the refining process reduces the lipid content, the C signals were larger. On the other hand, ozonized samples have larger amounts of oxygen elements, suggesting that the introduction of oxygen atoms during the hydroxyl oxidation reaction effectively occurs. Similar effects were observed by other authors during ozone-induced oxidation in OSA waxy rice starch [7].

**Figure 4:**
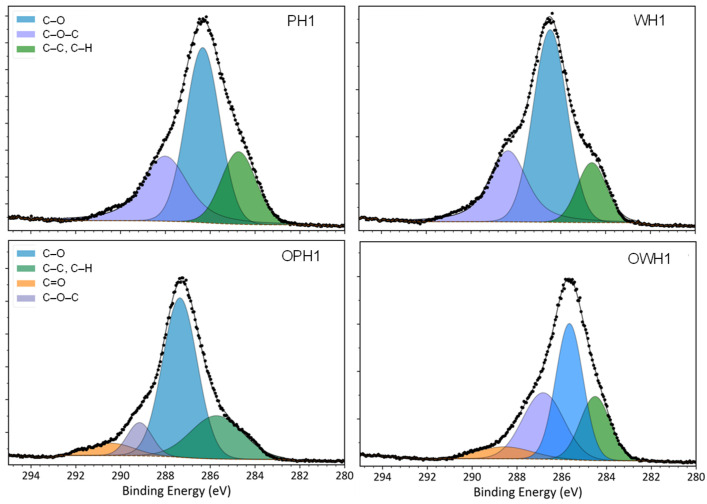
XPS spectra of polished (P) and whole (W) sorghum flour extrudates. High-resolution carbon signal peak deconvolution spectra of flours extruded with distilled water and ozonated water (O).

**Figure 5:**
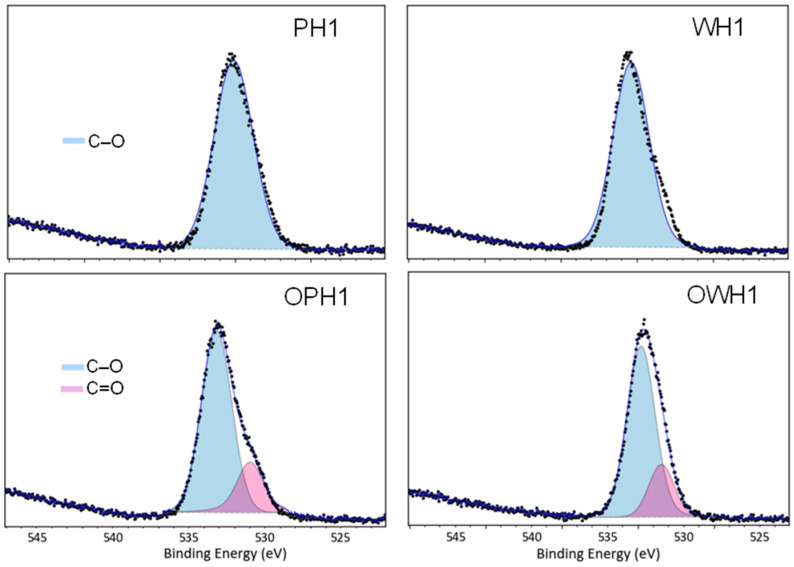
High-resolution oxygen signal XPS spectra of polished (P) and whole (W) sorghum flour extrudates and peak deconvolution spectra of flours extruded with distilled water and ozonated water (O).

Figure 4 and Figure 5 illustrate the C and O XPS high-resolution spectra of four samples with the same extrusion process conditions but different feeding materials.

The spectrum of C1s in both non-ozonated samples (PH1 and WH1) denote three characteristics peaks: the peak at 284.8 eV corresponding to C–C and C–H single bonds, the C–O single bond peak at 286.3 eV, and the C–O–C peak at 287.7 eV. In turn, the ozone-treated samples (OPH1 and OWH1) displayed a different peak near 288.7 eV belonging to the C1s spectra, which belong to the carbon of a C=O double bond. This result suggested that the starch had been successfully oxidized when ozonized water was used, but no significant differences were detected between polished and whole flour.

The O1s high-resolution spectrum of PH1 and WH1 samples showed one peak at 532.6 eV, which corresponds to a C–O single bond (Figure 5). In ozone-treated samples, a C=O double bond signal at 530.8 eV was observed. This peak indicates that the starch had been successfully oxidized, and consequently, carbonyl and carboxyl groups were present.

An X-ray photoelectron spectroscopy (XPS) analysis of ozone-added extrudates reveals significant modifications in surface chemistry. Both C and O spectra show that ozone treatment promotes the oxidation of the carbohydrate matrix. When treated with ozone, starch contained a higher proportion of oxygen, suggesting that hydroxyl groups were oxidized into carbonyl and carboxyl groups [2]. Repulsive interactions among oxidized starch groups can facilitate the penetration of water into starch particles [7]. This increase in oxidized carbon signals is consistent with the changes in the functional properties previously discussed. The molecular interactions were reflected at the macromolecular scale in both the RVA pasting profiles and the water absorption index (WAI), as previously discussed.

#### 3.4.2. Water Solubility and Absorption Indexes

**Figure 6:**
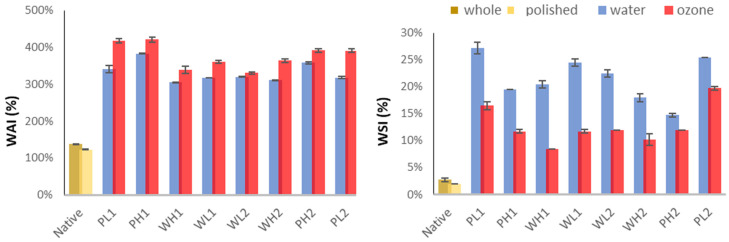
Water absorption (WAI) and water solubility indexes (WSI) of polished (P) and whole (W) sorghum flours.

The water absorption index (WAI) and water solubility index (WSI) were strongly influenced by flour type, ozone treatment, and extrusion conditions (Figure 6). Polished flour exhibited higher WAI than whole flour due to greater starch availability, whereas whole flour had lower solubility (WSI) because the fibers restricted starch release. Ozone treatment increased WAI in both flours by oxidizing starch and proteins, enhancing the water-binding capacity, but reduced WSI—probably due to protein aggregation and the complex formation with lipids. Higher extrusion temperatures (160 °C) increased WAI by promoting starch gelatinization but decreased WSI due to molecular degradation. Conversely, lower temperatures (110–140 °C) preserved solubility but limited water absorption. These phenomena, which imply starch gelatinization, can cause significant structural damage and dextrinization, leading to reduced water holding capacity; this has been extensively studied and reviewed [4]. Increased water flow rates enhanced WAI by helping starch hydration but reduced WSI due to milder shear forces.

Regarding the water flow rate, WAI was generally increased as moisture increased (Figure 6), primarily because water acts as a lubricant, facilitating starch granule swelling and gelatinization. Low moisture content intensifies shear and friction forces, which can lead to starch fragmentation, reducing WAI. Liu et al. [15] investigated the optimization of the extrusion process in black wheat flours and found that WSI values decrease with an increase in water content. They observed the same inverse relationship between the water flow rate and WSI values as those reported in Figure 6.

#### 3.4.3. Flour Color

Processing conditions had a slight but significant effect on the color (L*, a*, b*) of extruded sorghum flours, where higher changes were presented by the L* parameter (Table 5). The luminosity values of extruded whole flours ranged between 41.7 and 45.9, whereas polished flour ranged between 49.0 and 54.7. Polished flours exhibited greater lightness (higher L) compared to whole flours, which retained darker hues from their bran components. Sorghum flours, like other colored cereals, showed a negative correlation between L and the polyphenol and bran content [16,22].

The chroma value a* showed similar values for all samples with an average of 9.5; however, b* changed between 11.2 and 13.2. Higher extrusion temperatures (160 °C) promoted Maillard reactions and caramelization, decreasing L (darkening) while increasing b* (yellowness). Lower temperatures (110–140 °C) preserved lighter colors but with less intense chromaticity. Similar results were reviewed by Yadav et al. [28] for millets, a crop comparable to sorghum in use and composition.

Color changes in extruded sorghum flours were primarily driven by thermal reactions and oxidative effects of ozone. Then, total color difference was applied between samples treated under the same conditions but that differed regarding ozone presence (Table 6). Lower differences were observed in most samples with higher humidity. Increased water flow rates mitigated thermal degradation effects, helping maintain original color values by reducing residence time at high temperatures.

In summary, ozone treatment caused slight bleaching effects in polished flours (increased L), but this was less pronounced in whole flours mainly due to their higher tannin and fiber contents. It was reported that ozonation produced only a marginal improvement in flour color, evidenced by the minimal difference between the color values of polished and unpolished flours [29]. Other research studies also agree that treating sorghum flour with ozone noticeably raises its lightness (L value) and reduces its yellowness (b value) [30].

These findings demonstrate that product color can be modulated by selecting a suitable flour type (polished for lightness, whole for darker hues), ozone treatment (mild bleaching), and processing conditions (temperature vs. water flow rate balance).

#### 3.4.4. Viscosity Profiles

**Figure 7:**
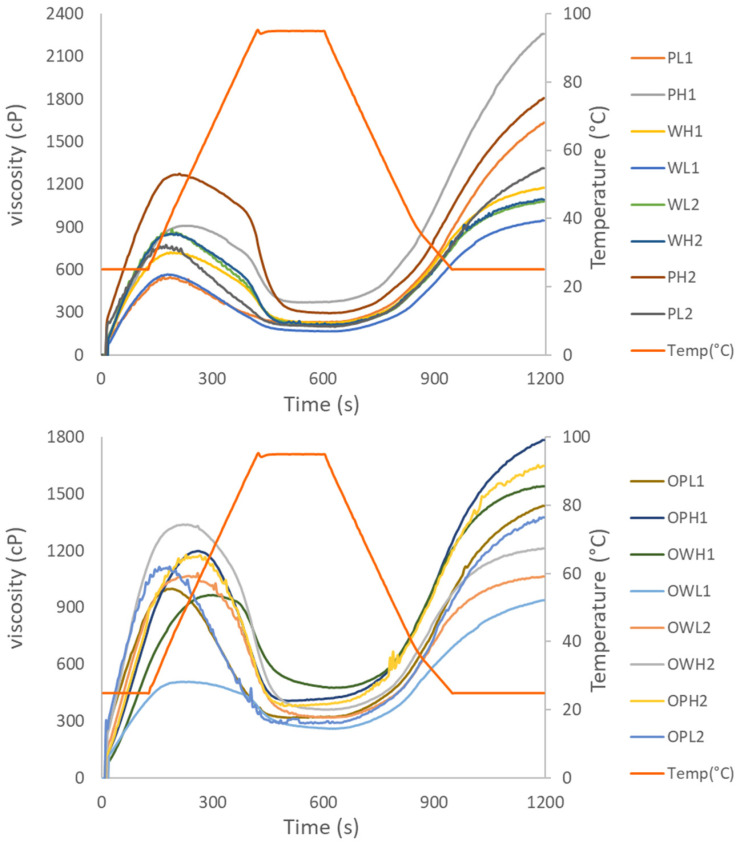
Rapid viscosity analyzer (RVA) pasting profile from polished (P) and whole (W) sorghum flours.

Rapid viscosity analyzer (RVA) profiles revealed distinct viscosity patterns influenced by processing conditions and ozone reactions (Figure 7). Polished flours showed a higher final viscosity (FV) and consistency peak (CP) than whole flours, a result of their greater starch content and gelatinization capacity. A notably high CP was seen in samples with elevated feed moisture content, which likely reduces mechanical stress on granules, thereby limiting their disruption. The FV serves as an indicator of starch retrogradation extent during cooling [31]; it decreased with higher barrel temperature, whereas it increased with greater feed moisture content and screw speed.

Ozonation increased the peak temperature (PT) and modestly affected the cold peak (CP), while reducing the final viscosity (FV) (Table 7). This reduction was most pronounced in polished flour, due to starch depolymerization. In contrast, whole flour exhibited greater viscosity stability, attributable to protective fiber–starch interactions. The observed decrease in PT and FV can likely be attributed to structural weakening and granule disintegration induced by ozonation [32]. Ozone treatment induces significant but variable changes in pasting profiles, primarily driven by the competing mechanisms of molecular depolymerization and oxidation-induced crosslinking. The results differ across botanical sources and ozone extension treatments. Peak viscosity (PV), or hot peak viscosity, is the maximum viscosity achieved during the heating phase of starch or flour suspension and is different to cold peak, but was still extensively studied. The PV decreases as the ozonation time increases, which is attributed to the cleavage of glycosidic bonds, or significantly increases with long ozone exposure linked to the formation of carboxyl groups that promote water uptake or a dominant cross-linking effect that reinforces the starch matrix [6,7,33]. On the other hand, regarding the tendency of starch to retrograde upon cooling, FV decreasing by the introduction of bulky, electronegative carboxyl groups create electrostatic repulsion that impedes the reassociation of amylose chains [34].

Higher extrusion temperatures (160 °C) decreased the final viscosity (FV) and peak temperature (PT) via starch fragmentation and degradation. In contrast, moderate temperatures (140 °C) preserved viscosity by maintaining a partially intact granule structure, which allowed for controlled gelatinization without extensive breakdown. Higher water flow rates result in higher viscosity parameters by increasing shear-induced starch damage, particularly in polished flour. Higher moisture content provides sufficient water for starch gelatinization, causing extensive molecular degradation and higher CP and FV values.

Profiles proved that viscosity properties are governed by starch integrity and matrix interactions. Ozone and high temperatures promoted starch degradation, reducing viscosity, while whole flour’s fiber content mitigated these effects. Polished flour showed greater viscosity but was more susceptible to processing-induced changes. Optimal viscosity for specific applications (e.g., high for thickening, low for instant solubility) can be achieved by balancing ozone treatment, flour type, and extrusion parameters (temperature and water flow rate). These insights enable the tailored modification of sorghum flours for diverse food formulations.

### 3.5. In Vitro Digestion of Sorghum Extrudates

**Figure 8:**
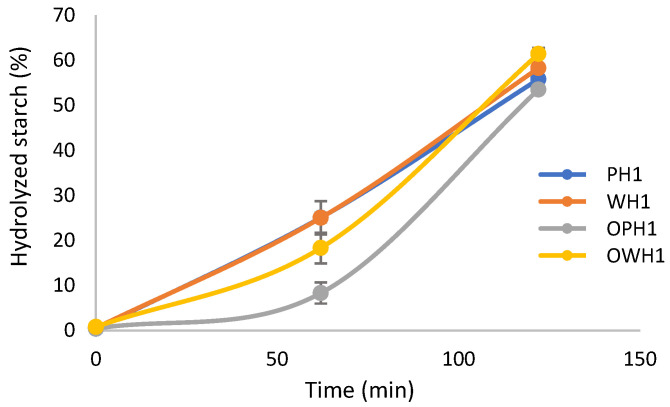
Starch hydrolysis quantified in the supernatant after in vitro digestion.

During in vitro digestion, starch and protein hydrolysis were monitored. The evolution of starch hydrolysis was analyzed after the oral, gastric, and intestinal endpoints (Figure 8). Both non-ozonized flours showed higher starch hydrolysis values (~25%) at the gastric phase than ozonized whole grain sorghum flour (18.3%) or ozonized polished flour (8.3%). Mild ozonation treatment decreased digestibility in sorghum ozonated starch, which is attributed to the change in the functional groups [35]. About 58% of starch was hydrolyzed after digestion in all samples, indicating that polishing or ozonizing sorghum flour before extrusion did not affect starch susceptibility to digestive enzymes at the end of in vitro digestion. Sorghum starch digestion is dependent on extrusion conditions [36], a constant in the four analyzed samples. A study on corn extruded snacks supplemented with rice bran demonstrated that the inclusion of dietary fibers can significantly influence the rate of starch digestion [26]. The presence of fibers in these snacks lowers the starch digestion rate during the initial phase of the assay. However, as the assay progressed, all samples (with and without rice bran) eventually reached similar levels of starch digestion by the end of the test. These findings suggest that fiber supplementation can modulate the early stages of starch hydrolysis, but its impact diminishes over time.

**Figure 9:**
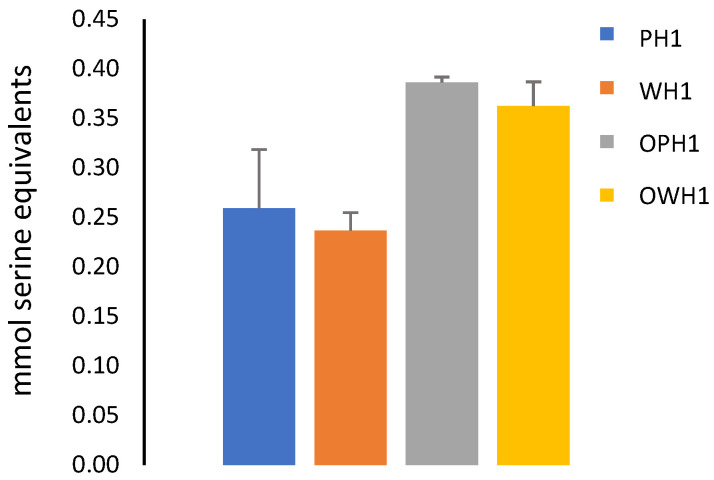
Absolute values of mmol serine equivalents corresponding to free amino groups (0.5 g protein) quantified in the supernatant after in vitro digestion.

During the gastric phase, the hydrolysis of proteins that prevent enzyme access to starch directly influences its digestion. As a result, we quantified amino acids and small peptides released from extruded samples after digestion (Figure 9). During in vitro digestion, increased protein hydrolysis in ozonized samples exposes starch to digestive enzymes, but its altered structure by ozone prevails and slows down starch hydrolysis at the gastric step (particularly for whole ozonized sorghum flour) until all samples reach a similar endpoint at the intestinal phase. That is, the addition of amylase during the intestinal step increased the rate of starch hydrolysis in ozonized samples due to the earlier degradation of proteins. These results agree with those of Xiang et al. [37], who highlighted that the differences in the structure of the protein–starch matrix affect starch susceptibility to hydrolysis during digestion.

Extrusion has been shown to increase sorghum protein hydrolysis [1], and the present results support this observation, with approximately 3.5 times more amino acids quantified than de Sousa et al. [38], who also studied sorghum flour. Then, the reactive extrusion ozonation seemed to enhance protein availability, overcoming a major issue in sorghum consumption. Additionally, the starch hydrolysis of sorghum extrudates has not been extensively studied, which highlights the novelty of this present research. Therefore, the use of ozone treatment on sorghum flours for extrudate production may be linked to enhanced protein digestibility and a reduction in the rate of starch hydrolysis in extruded samples.

## 4. Conclusions

The reactive extrusion carried out with a twin extruder coupled with saturated ozone solution in feed water significantly changes the extrudate and resultant flour properties. Extruder process parameters were affected, and conditions for minimal SME involved the ozone-assisted extrusion of polished flour at elevated temperatures (160 °C) and higher water flow rates, whereas whole flour benefited from lower temperatures (140 °C) to balance energy input and functional properties. Ozone treatment alters the structure of extrudates, especially in polished flour, where it increases density but decreases expansion and fracture force. These changes occur due to starch degradation and diminished polymer interactions, which weaken the extrudate.

Water-related functional properties were also changed with ozone addition and visibly governed by starch accessibility and structural modifications. Ozone improved WAI but reduced WSI by promoting aggregation, whereas whole flour fiber content restricted solubility. Higher temperatures and ozone presence promoted starch degradation, reducing viscosity profile parameters, but less of an effect was observed in whole flours. In vitro digestion results indicate that ozonation improved protein digestibility while also decreasing the rate of starch hydrolysis in extruded samples, especially in the early stages.

Sorghum extrudates developed in this research differ from extrudate products available on the market mainly because commercial products are characterized by high and fast starch digestibility, low protein content, and high fracture force. Future work should focus on the sensorial analysis and in vivo bioavailability studies of extrudates.

## Figures and Tables

**Table 1:** Temperature profile of the different zones.

Temperature Profile (°C)								
N°	Die	Zone 8	Zone 7	Zone 6	Zone 5	Zone 4	Zone 3	Zone 2
1	140	140	120	110	100	90	60	30
2	160	160	120	110	100	90	60	30

**Table 2:** Sorghum flour proximal composition and total phenolic content (TPC).

Raw Materials	Humidity	Carbohydrates *	Proteins	Lipids	Ash	TPC **
Polished Flour	4.03 ± 0.11	87.14	10.97 ± 0.02	0.29 ± 0.00	1.60 ± 0.01	24.2 ± 0.5
Whole flour	4.80 ± 0.06	85.89	10.73 ± 0.07	1.63 ± 0.66	1.75 ± 0.02	39.8 ± 2.1

Values are expressed as g/100 g of sample. * Calculated by difference. ** mg of gallic acid per 100 g of flour (dry basis).

**Table 3:** Extrusion process parameters for sorghum flours.

Sample	Feeding Moisture (%)	Final Zone Temperature (°C)	Torque (Nm)	Die Temperature (°C)	Die Pressure (Bar)	SME (W h kg^−1^)
PL1	20	140	5.6	135	10.5	227.50
PH1	23	140	3.6	135	7.0	146.25
WH1	23	140	5.0	137	7.5	203.13
WL1	20	140	7.6	142	13.5	308.75
WL2	20	160	4.6	150	8.0	186.88
WH2	23	160	5.8	152	8.0	235.63
PH2	23	160	3.0	149	4.0	121.88
PL2	20	160	6.1	154	9.5	247.81
OPL1	20	140	4.4	136	10	178.75
OPH1	23	140	3.6	132	9	146.25
OWH1	23	140	5.4	131	14	219.38
OWL1	20	140	6.2	136	15	251.88
OWL2	20	160	5.1	143	10	207.19
OWH2	23	160	4.6	147	9	186.88
OPH2	23	160	3.8	146	8	154.38
OPL2	20	160	5.2	148	12	211.25

Reference for samples. O: ozonized. P and W: polished and whole flours. L and H: low and high water flow rates, respectively. 1 and 2: low and high-temperature extrusion profiles, respectively.

**Table 4:** Structural properties of sorghum flour extrudates.

Sample	Density (g cm^−3^)	Expansion Index	Fracture Force (N)
PL1	0.45 ± 0.03 ^c^	1.72 ± 0.10 ^a^	12.8 ± 1.7 ^e^
PH1	0.52 ± 0.01 ^d^	1.94 ± 0.12 ^b^	11.4 ± 3.2 ^d^
WH1	0.45 ± 0.04 ^c^	1.83 ± 0.05 ^a^	12.3 ± 2.5 ^e^
WL1	0.35 ± 0.02 ^b^	2.16 ± 0.14 ^c^	11.8 ± 1.8 ^d^
WL2	0.49 ± 0.06 ^d^	1.83 ± 0.06 ^a^	6.1 ± 1.0 ^a^
WH2	0.56 ± 0.08 ^d^	1.77 ± 0.09 ^a^	9.6 ± 1.9 ^c^
PH2	0.66 ± 0.05 ^e^	1.99 ± 0.15 ^b^	9.9 ± 1.3 ^c^
PL2	0.31 ± 0.02 ^a^	2.38 ± 0.12 ^d^	13.6 ± 1.8 ^f^
OPL1	0.49 ± 0.04 ^d^	2.00 ± 0.04 ^b^	10.5 ± 2.7 ^d^
OPH1	0.53 ± 0.08 ^d^	1.70 ± 0.18 ^a^	18.3 ± 1.6 ^i^
OWH1	0.46 ± 0.04 ^c^	2.20 ± 0.20 ^c^	14.7 ± 2.2 ^g^
OWL1	0.37 ± 0.04 ^b^	2.00 ± 0.13 ^b^	16.6 ± 1.4 ^h^
OWL2	0.39 ± 0.03 ^b^	1.84 ± 0.05 ^a^	7.4 ± 1.6 ^b^
OWH2	0.45 ± 0.01 ^c^	2.28 ± 0.10 ^c^	8.6 ± 1.3 ^b^
OPH2	0.50 ± 0.08 ^d^	2.25 ± 0.11 ^c^	8.2 ± 1.3 ^b^
OPL2	0.37 ± 0.03 ^b^	2.21 ± 0.07 ^c^	10.9 ± 2.0 ^d^

Reference for samples. O: ozonized. P and W: polished and whole flours. L and H: low and high water flow rates, respectively. 1 and 2: low and high-temperature extrusion profiles, respectively. Different superscript letters within each column indicate significant difference between samples (*p* < 0.05).

**Table 5:** Color parameters of flours from sorghum extrudates.

Sample	L*	a*	b*
PL1	54.57 ± 0.65 ^d^	9.30 ± 0.07 ^b^	13.03 ± 0.05 ^c^
PH1	53.26 ± 3.08 ^d^	9.33 ± 0.32 ^b^	13.17 ± 0.18 ^c^
WH1	45.26 ± 1.38 ^c^	9.51 ± 0.15 ^b^	11.55 ± 0.21 ^a^
WL1	43.22 ± 0.84 ^b^	9.57 ± 0.49 ^b^	11.95 ± 0.86 ^b^
WL2	45.96 ± 0.79 ^c^	9.83 ± 0.19 ^b^	12.23 ± 0.31 ^b^
WH2	41.78 ± 1.66 ^a^	9.61 ± 0.41 ^b^	11.27 ± 0.58 ^a^
PH2	54.18 ± 2.40 ^d^	9.18 ± 0.20 ^b^	12.68 ± 0.15 ^c^
PL2	49.05 ± 0.96 ^c^	9.57 ± 0.43 ^b^	12.85 ± 0.60 ^c^
OPL1	45.98 ± 2.20 ^c^	9.10 ± 0.39 ^b^	11.82 ± 0.59 ^a^
OPH1	52.06 ± 0.30 ^d^	8.97 ± 0.13 ^b^	12.32 ± 0.18 ^b^
OWH1	45.84 ± 0.95 ^c^	9.81 ± 0.14 ^b^	12.30 ± 0.12 ^b^
OWL1	40.03 ± 0.95 ^a^	9.31 ± 0.44 ^b^	11.01 ± 0.41 ^a^
OWL2	44.32 ± 1.84 ^b^	9.87 ± 0.08 ^b^	11.86 ± 0.29 ^b^
OWH2	47.38 ± 0.07 ^c^	9.53 ± 0.13 ^b^	12.16 ± 0.03 ^b^
OPH2	55.07 ± 0.28 ^d^	8.73 ± 0.05 ^a^	12.16 ± 0.13 ^b^
OPL2	53.64 ± 0.13 ^d^	9.37 ± 0.12 ^b^	12.46 ± 0.10 ^b^

Reference for samples. O: ozonized. P and W: polished and whole flours. L and H: low and high water flow rates, respectively. 1 and 2: low and high-temperature extrusion profiles, respectively. Different superscript letters within each column indicate significant difference between samples (*p* < 0.05).

**Table 6:** Total color difference (ΔE) between samples extruded with and without ozone under the same process conditions.

Samples	ΔE
PL1 vs. OPL1	8.68
PH1 vs. OPH1	1.51
PH2 vs. OPH2	1.12
PL2 vs. OPL2	4.61
WH1 vs. OWH1	0.99
WL1 vs. OWL1	3.33
WL2 vs. OWL2	1.69
WH2 vs. OWH2	5.66

Reference for samples. O: ozonized. P and W: polished and whole flours. L and H: low and high water flow rates, respectively: 1 and 2: low and high-temperature extrusion profiles, respectively.

**Table 7:** Rapid viscosity analyzer (RVA) profile main parameters of flours from sorghum extrudates.

Sample	Cold Peak (cP)	Final Viscosity (cP)	Peak Temp. (°C)
PL1	564 ± 14 ^a^	1876 ± 24 ^e^	36 ± 4 ^a^
PH1	741 ± 24 ^b^	2238 ± 23 ^f^	48 ± 3 ^b^
WH1	703 ± 28 ^b^	1156 ± 20 ^b^	41 ± 2 ^a^
WL1	569 ± 10 ^a^	921 ± 26 ^a^	38 ± 1 ^a^
WL2	865 ± 24 ^c^	1066 ± 11 ^b^	39 ± 1 ^a^
WH2	845 ± 11 ^c^	996 ± 94 ^a^	39 ± 2 ^a^
PH2	1502 ± 32 ^f^	1968 ± 16 ^e^	43 ± 3 ^b^
PL2	760 ± 17 ^b^	1423 ± 11 ^c^	39 ± 1 ^a^
OPL1	1008 ± 11 ^d^	1444 ± 7 ^c^	41 ± 1 ^a^
OPH1	1186 ± 16 ^e^	1755 ± 27 ^e^	56 ± 3 ^c^
OWH1	942 ± 33 ^d^	1559 ± 20 ^d^	54 ± 4 ^c^
OWL1	878 ± 20 ^c^	1042 ± 3 ^a^	52 ± 1 ^c^
OWL2	1072 ± 17 ^d^	1048 ± 17 ^a^	46 ± 3 ^b^
OWH2	1251 ± 12 ^e^	1207 ± 4 ^b^	47 ± 7 ^b^
OPH2	1265 ± 12 ^e^	1645 ± 10 ^d^	54 ± 1 ^c^
OPL2	1131 ± 20 ^d^	1374 ± 30 ^c^	36 ± 3 ^a^

Reference for samples. O: ozonized. P and W: polished and whole flours. L and H: low and high water flow rates, respectively. 1 and 2: low and high-temperature extrusion profiles, respectively. Different superscript letters within each column indicate significant difference between samples (*p* < 0.05).

## Data Availability

The original contributions presented in this study are included in the article. Further inquiries can be directed to the corresponding author.

## Abbreviations

The following abbreviations are used in this manuscript:

ANOVA	Analysis of Variance
AOAC	Association of Official Analytical Chemists
BDV	Breakdown Viscosity
CP	Cold Peak
CT	Cold Peak (Initial Hydration)
DNS	3,5-Dinitrosalicylic Acid
El	Expansion Index
FV	Final Viscosity
GA	Gallic Acid
HTST	High-Temperature, Short-Time
L/D	Length-to-Diameter
OPA	o-Phthaldialdehyde
PT	Peak Temperature
PV	Peak Viscosity
RVA	Rapid Visco Analyzer
SBV	Setback Viscosity
SGF	Simulated Gastric Fluid
SIF	Simulated Intestinal Fluid
SME	Specific Mechanical Energy
SSF	Simulated Salivary Fluid
TPC	Total Polyphenol Content
WAI	Water Absorption Index
WSI	Water Solubility Index
XPS	X-ray Photoelectron Spectroscopy
